# Sleep deprivation disrupts striatal anti-apoptotic responses in 6-hydroxy dopamine-lesioned parkinsonian rats

**DOI:** 10.22038/ijbms.2018.28546.6919

**Published:** 2018-12

**Authors:** Nahid Ahmadian, Javad Mahmoudi, Mahnaz Talebi, Leila Molavi, Saeed Sadigh-Eteghad, Egill Rostrup, Mojtaba Ziaee

**Affiliations:** 1 Neurosciences Research Center, Tabriz University of Medical Sciences, Tabriz, Iran; 2 Department of Molecular Medicine, Faculty of Advanced Biomedical Sciences, Tabriz, Iran; 3 Pharmaceutical Biotechnology Department, Faculty of Pharmacy, Tabriz University of Medical Sciences, Tabriz, Iran; 4 Mental Health Centre Glostrup, University of Copenhagen, Glostrup, Denmark; 5 Cardiovascular Research Center, Tabriz University of Medical Sciences, Tabriz, Iran

**Keywords:** Bcl-2, BDNF, Bax, Parkinson’s disease, Sleep deprivation, 6-Hydroxy dopamine

## Abstract

**Objective(s):**

The present study was conducted to examine the effect of sleep deprivation (SD) on the anti-apoptotic pathways in Parkinsonian rats.

**Materials and Methods:**

Male Wistar rats (n = 40) were assigned to four groups (10 animals each): sham surgery (Sham), 6-hydroxydopamine (6-OHDA)-lesioned (OH), 6-OHDA-lesioned plus grid control (OH+GC), 6-OHDA-lesioned plus SD (OH+SD). Parkinson’s disease (PD) model was induced by the unilateral intra-striatal infusion of 6-OHDA (10 µg/rat). SD (4 hr/day, for 14 days) was induced using a multiple platforms water tank. On the last day of interventions, animals were subjected to open field test for horizontal motor performance assessment. Also, brain-derived neurotrophic factor (BDNF), Bcl-2 and Bax were assessed in the striatum of study groups.

**Results:**

SD obscured the motor deficits of PD animals observed in open field test. BDNF level and Bcl2/Bax ratio significantly increased in the OH group, and SD reduced their levels in the PD animals.

**Conclusion:**

SD suppressed the anti-apoptotic compensatory responses in the striatum; therefore, it may accelerate continual neuronal cell death in PD.

## Introduction

Parkinson’s disease (PD) is a progressive and disabling neurodegenerative disorder affecting nearly 1-2% of elderlies. It results in dysfunction of dopaminergic (DAergic) neurons in the nigrostriatal system. This accounts for major PD symptoms including resting tremor, muscle rigidity, and bradykinesia (1). From pathological view, excitotoxicity, mitochondrial impairment, oxidative stress and decreased antioxidant capacity occur during PD leading to apoptotic cell death in the nigrostriatal pathway (2, 3). Degeneration of DAergic neurons progresses silently during PD until clinical signs appear in the late and advanced clinical phases (4). In fact, motor features of PD do not appear until 30% of substantia nigra pars compacta neurons and nearly 80% of striatal dopamine (DA) contents are lost (5). It has been long proposed that the activation of compensatory functions either within or outside the basal ganglia structures may contribute to the gradual progression of PD (6). In this phenomenon, some of the unaffected neuronal systems become able to compensate the deficits occurred in PD (7). The non-linear relationship between DAergic neurons destruction and behavioral dysfunction suggests that adaptive neurochemical alterations may have role to stabilize DA-mediated neuronal homeostasis in PD (8). Beside the function of different enzymes and transporters that are necessary for synaptic activity of DAergic neurons (5), molecules such as brain-derived neurotrophic factor (BDNF) as well as anti- and pro- apoptotic proteins (i.e. Bax and Bcl-2 proteins, respectively) are crucial in the viability of these neurons (9, 10). The majority of these components are influenced by adaptive reactions to reduce the effect of DAergic neuronal loss on the motor signs at least in the pre-clinical stage of the disease (8, 11). Thus, an increase in BDNF and Bcl-2/Bax ratio has been shown to delay progressive cell loss and spare remaining DAergic neurons. This restores their lost functions in regulation of DA release and recovers behavioral functions (12, 13). Prominent diurnal and nocturnal sleep disturbances including excessive daytime sleepiness, parasomnias and insomnia are frequently reported in PD and nearly all of affected patients experience these abnormalities early in the disease onset (1). Among these, insomnia that is known as the most common sleep complication results in reduction of quality of life and increased burden of the illness (1, 14). Also, the symptoms of insomnia are found in two-third of the patients (14) in whom sleep initiation or maintaining is cumbersome (also called sleep onset insomnia and sleep fragmentation). These patients may also experience un-refreshing sleep in the morning (14-16). Insomnia or sleep deprivation (SD) affects the overall activity of neuronal cells and may increase neuronal vulnerability to the neurodegenerative insults (17). Indeed, sleep as a homeostatic procedure regulates normal body functions and in particular it is critical for restoration, replenishment and reorganization of the brain functions (18). Normal neuronal activity causes deposition of active and potentially toxic biomolecules during awakeness, and sleep is thought to eliminate such products (19, 20). Therefore, sleep is able to repair and regenerate some regions of brain to prevent neuronal dysfunction (19, 21). Given the possible negative effects of SD on normal neuronal function and the prevalence of different types of sleep disorders in patient with PD, we hypothesized that induction of chronic SD in rats with 6-hydroxy-dopamine (6-OHDA) lesion may attenuate compensatory responses. 6-OHDA is a hydroxylated analogue of DA that is used to model PD pathology in rats (22). It is a highly oxidizable compound that undergoes auto-oxidation and produces a series of reactive oxygen spices (ROS) (23). Therefore, 6-OHDA is able to cause ROS-dependent apoptotic cell death in DAergic neurons (24) and following intra-striatal infusion, it degenerates nigral DAergic neurons over a 14-day period (25). In this study, beside behavioral assessment, we evaluated the alterations of Bax, Bcl-2 and BDNF in the striatum of 6-OHDA-lesioned rat.

## Materials and Methods

***Animals and experimental design***

Male Wistar rats (Laboratory animal unit of Tabriz University of Medical Sciences) (180-220 g) were used in this study. Rats were kept in standard polypropylene cages and maintained at constant temperature of 23 ^°^C with free access to food and tap water. Animals were housed at a 12 hr light/dark schedule (light at 7:00 AM). All experimentations were performed during the light cycle. In order to prevent occurrence of social instability, before initiation of the experiment, rats were kept in the same cages for 15 days to stabilize a social hierarchy within the group. This study was confirmed by the Ethical Committee of the Tabriz University of Medical Sciences (TBZMED.REC.1394.838), and all experimental procedures were performed in accordance with the National Research Council (US) Committee for the Update of the Guide for the Care and Use of Laboratory Animals (Guide for the Care and Use of Laboratory Animals, 8^th^ edition, 2011). 

**Figure 1:**
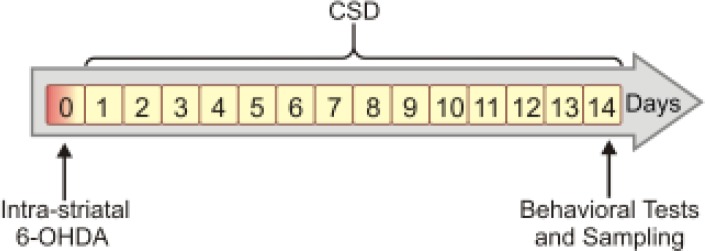
Time course of intra-striatal injection of 6-hydroxydopamine (6-OHDA) and submitting animals in OH+sleep deprivation (SD) group to SD protocol

Animals were randomly allocated to one of the following groups (n=10 each): sham surgery (Sham), 6-OHDA-lesioned (OH), 6-OHDA-lesioned plus chronic SD (OH+SD), and 6-OHDA-lesioned plus grid control (OH+GC). Rats in the sham group underwent stereotaxic surgery and received 6-OHDA vehicle. OH, OH+SD and OH+GC groups were subjected to the intra-striatal injection of 6-OHDA. Rats in the sham and OH groups were kept in their home cages throughout the study period. Using a modified multiple platforms (MMP) tank, rats in the OH+SD group were subjected to the SD protocol; 4 hr/day for 14 days (Figure 1). The OH+GC rats were placed on a grid floor inside the water tank.

**Figure 2:**
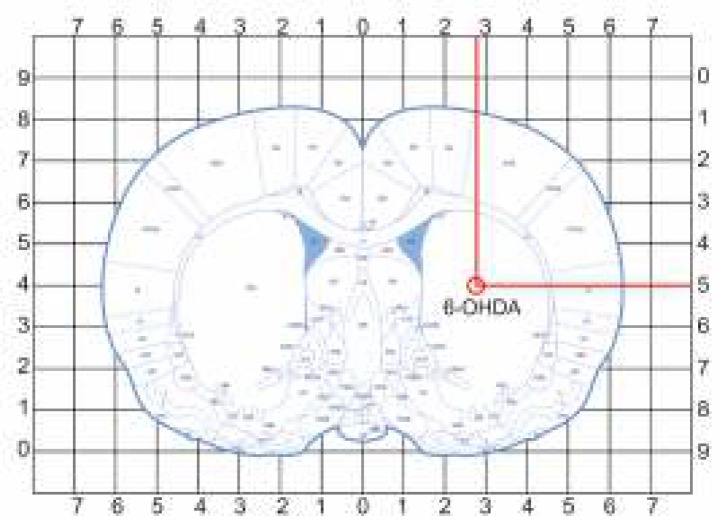
Intra-striatal injection site of 6-hydroxydopamine (6-OHDA) according to its representative stereotaxic coordination in the Paxinos & Watson atlas (26)

**Figure 3:**
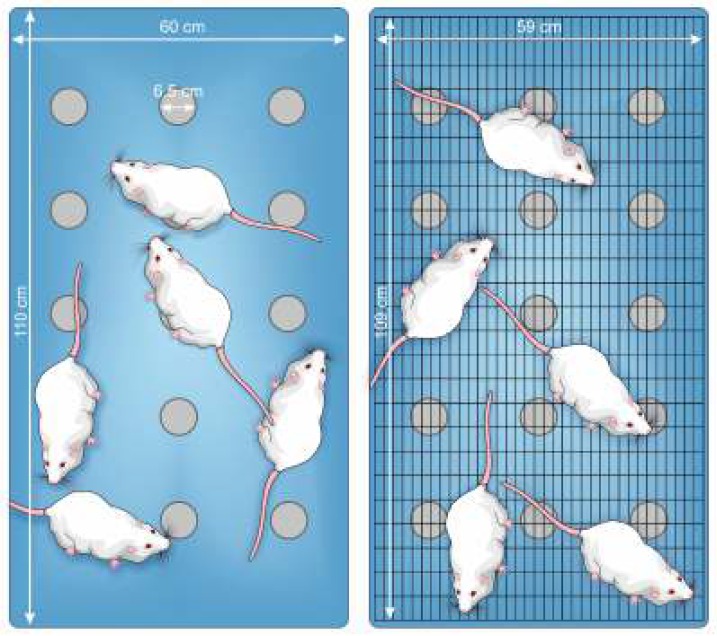
Schematic top view of modified multiple platform tanks used for rats in OH+SD (left panel) and OH+GC (right panel) groups. (OH, 6-hydroxydopamine; GC, grid control; SD, sleep deprivation)

**Figure 4:**
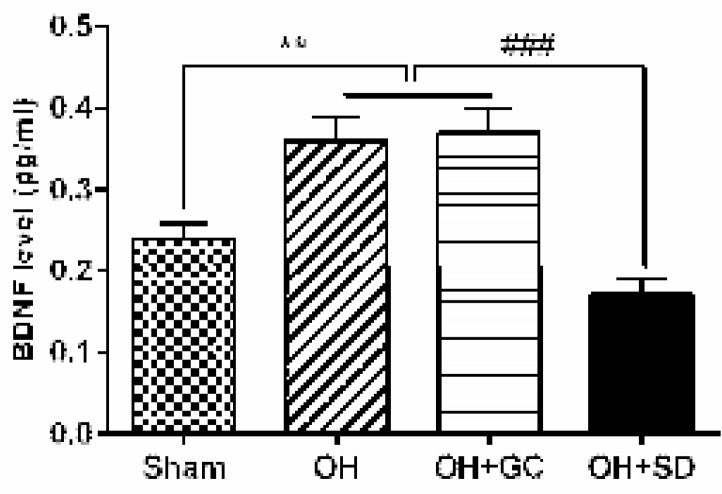
Striatal expression of brain-derived neurotrophic factor (BDNF) of sham, OH, OH+GC and OH+SD rats. Each bar represents the mean±SEM. (n=10). **P<0.01 compared with the sham and ###*P<*0.001 compared with the OH and OH+GC groups. (OH, 6-hydroxydopamine; GC, grid control; SD, sleep deprivation)

**Figure 5:**
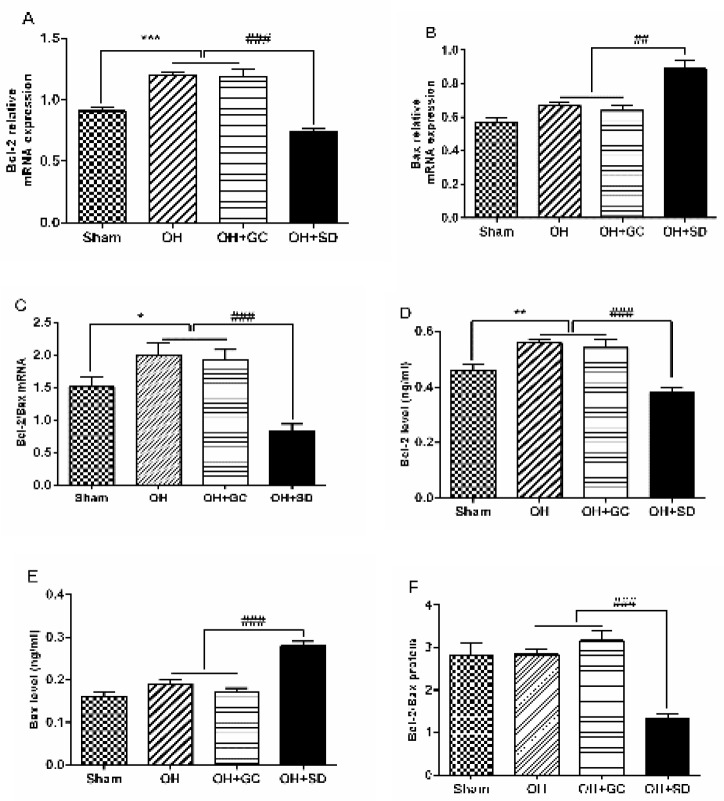
Striatal Bcl-2 and Bax mRNA (A and B) and protein (D and E) expression as well as Bcl-2/Bax ratios at mRNA (C) and protein levels (F) among study groups. Each bar represents the mean ± SEM. (n=10). **P<0.01, and *P<0.05 compared with the sham and ##*P<*0.01 compared OH and OH+GC groups. (OH, 6-hydroxydopamine; GC, grid control; SD, sleep deprivation)

**Figure 6:**
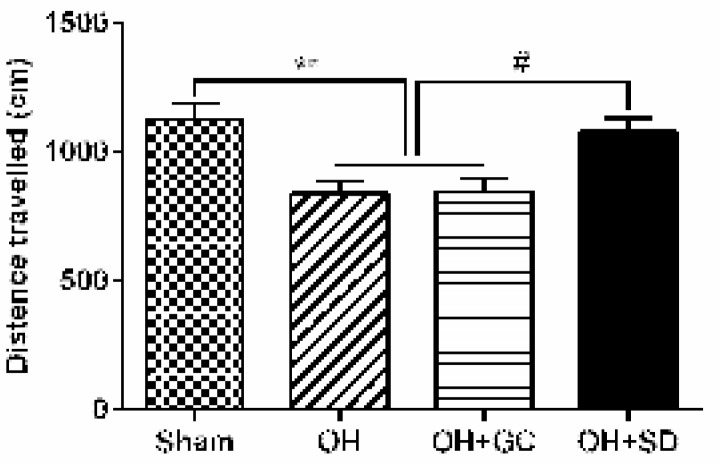
The mean total distance travelled in the open field by control, sham, OH, OH+GC and OH+SD rats. Each bar represents the mean± SEM. (n=10). **P<0.01 compared with the sham group and #*P<*0.05 compared with the OH and OH+GC groups. (OH, 6-hydroxydopamine; GC, grid control; SD, sleep deprivation)

**Table 1:** Specifications of the primers used for qRT-PCR

**Gene**	**Primer (5’3’)**	**Product size (bp)**
**β-actin**	Forward: AAGTTCAACGGCACAGTCAAGG Reverse: CATACTCAGCACCAGCATCACC	121
**Bcl-2**	Forward: GAGCGTCAACAGGGAGATGTC Reverse: TGCCGGTTCAGGTACTCAGTC	72
**Bax**	Forward: GACTCCCCCCGAGAGGTCTT Reverse: ACAGGGCCTTGAGCACCAGTT	121

***6-OHDA lesion***

Unilateral lesions of the right striatum was induced by the stereotaxic infusion of 6-OHDA hydrobromide (10 μg/rat in 2 μl containing 0.9% saline with 0.02% w/v ascorbic acid; Sigma Chemical Co, USA) under ketamine and xylazine anesthesia (80 and 5 mg/kg, intraperitoneally, respectively). The coordinates for this site were determined according to the rat brain stereotaxic coordinates: anteroposterior from bregma (AP) = 0.4 mm, mediolateral from the midline (ML)= 2.8 mm and dorsoventral from the skull (DV)= -5 mm (26) (Figure 2). Desipramine HCl (25 mg/kg; Sigma Chemical Co, USA) was injected intraperitoneally 30 min before the intra-striatal microinfusion in order to limit uptake of neurotoxin into the noradrenergic neurons and their subsequent degeneration (27). Rats in the sham group was injected with 2 μl vehicle of 6-OHDA (0.9% saline containing 0.02% (w/v) ascorbic acid) into the same site.

***SD protocol***

SD was induced by MMP method in which the groups of 5 animals (from the same cage) were put in the MMP water tank (110 cm length, 60 cm width, and 40 cm height). The tank contained 15 platforms (8 cm high and cm 6.5 diameters), horizontally and vertically spaced apart by 13 cm and 10 cm (edge to edge), respectively and arranged in 3 rows such that did not limit rats movement from one platform to another. The tank was filled with water (24 ± 1 ^°^C) until the water surface reached 1 cm below the edge of the platforms. During the SD period, rats had access to unlimited food and water, which had been provided through hung bottles and pellets boxes. Upon entering into rapid eye movement (REM) sleep, rats lose their postural tone and contact with or drop into the water (28, 29). Therefore, MMP model only repeatedly prevents REM sleep without limiting animal’s movement and its exploratory behaviors (30, 31). Also, submitting animals to unfamiliar environment and SD protocol may be associated with a stressful experience (32). Therefore, in SD studies, a group of animals as GC group is used to assess probable stressful effects of the SD procedure (31). In this study, the rats in OH+GC group were submitted to the same procedure as the OH+SD rats except the platforms in water tank were covered with a stainless grid floor (109 cm length and 59 cm wide) to allow rats to sleep without falling into water (Figure 3). The animals in OH+SD and OH+GC groups were submitted to tank environment between 7:00 to 11:00 AM and then returned to their home cages.

***Open field test (OFT)***

For evaluation of horizontal motor performance, each rat was individually placed in the center of square open field arena (90× 90×45 cm) made of opaque black polyethylene. The locomotor activity of the animals during 5 min was videotaped by a ceiling-mounted video camera, and the total distance movement was analyzed using Noldus tracking software (Ethovision XT, Noldus Information Technology, Wageningen, Netherlands). After each test session, the arena was cleaned carefully with cotton wet with a 10% ethanol solution to remove the presence of any olfactory clues (33). 

***6-OHDA-induced lesion verification***

Rats with 6-OHDA lesion received an acute intraperitoneal injection of amphetamine (2.5 mg/kg) to confirm the efficacy of unilateral lesion of the striatum. Only rats exhibiting at least 5 full (360°) ipsilateral rotations per min were considered as Parkinsonian rats and included for sampling and data analysis (34).

***Sampling***

At the end of the behavioral experiment, the animals in different groups were euthanized and the brains were quickly removed from the cranium. In all groups, the right hemisphere was separated along the midline and striatum was dissected on ice. This hemisphere was selected for tissue collecting, because in the sham and OH groups, it underwent stereotaxic surgery. The collected striatum was rapidly frozen in the liquid nitrogen and then stored at -80 ^°^C for further processes.

***Isolation of RNA and real time PCR***

The alterations of Bcl-2 and Bax mRNA expression were determined using quantitative real time PCR (qRT-PCR). Total RNA of the right striatum was extracted using AccuZol solution (BioNeer, Korea), according to the manufacturers’ instructions. The quality and concentration of the isolated RNA were evaluated using agarose gel electrophoresis and NanoDrop (2000 UV-VIS spectrophotometer, Thermo Scientific, USA), and samples were stored at -80 ^°^C until the time of use. Complementary DNA (cDNA) was synthesized using 3 µg of total RNA by Accupower cyclescript RT premix (dn6) (BioNeer, Korea). qRT-PCR was performed by a LightCycler 96 - Roche system using 10 µl of SYBR Premix (RR820L, Takara, Japan), 0.4 µM of each primer (Table 1), 1 µl of cDNA template, and 8.6 µl of nuclease-free water. Thermocycler schedule was set as follows: The hot start step at 95 ^°^C for 3 min was followed by 45 cycles at 95 ^°^C for 10 sec, 62 ^°^C for 30 sec and 72 ^°^C for 20 sec. The relative mRNA expression levels of target genes in each sample were determined with ∆ Ct method (35), and β-actin was used as endogenous normalizer. 

***Enzyme-linked immunosorbent assay for BDNF, Bcl-2 and Bax***

For protein extraction, the striatum tissue was homogenized in the in lysis buffer containing 1 mmol KH_2_PO_4_ ,1 mmol KCl , 50 mmol Tris-HCl pH 7.4, 1 mmol ethylenediamine tetraacetic acid (EDTA), 1 mmol sodium fluoride (NaF), 1 mmol sodium orthovanadate (Na_3_VO_4_) and Triton X100 and protease inhibitors cocktail (Complete tablet; Roche), using polytron homogenizer (POLYTRON^®^ PT 10-35, Kinematica, Switzerland) on ice. The homogenized striatum was centrifuged at 4 ^°^C for 15 min at 10000×g to remove the insoluble materials form the specimen. Total protein concentration in the supernatant was determined using Bradford method. The striatal contents of BDNF, Bcl-2 and Bax were measured using commercially available ELISA kits (Elabscience Biotechnology Co, Ltd, USA) according to manufacturer’s recommendations. 

***Statistical analysis***

Statistical analysis was performed using Graph Pad Prism 6.01 (Graph Pad Software Inc., La Jolla, CA, USA). All data were expressed as mean ± SEM and were analyzed by one-way ANOVA followed by Tukey’s *post-hoc* test. *P*<0.05 was considered statistically significant.

## Results

***Striatal BDNF expression***

Striatal BDNF level was significantly higher in the OH group compared to the sham rats (*P*<0.01). BDNF decreased in the OH+SD group compared to the OH and OH+GC groups (*P*<0.001). No difference was found in the amount of this protein between the OH+GC and OH groups (Figure 4). 

***Bcl-2 and Bax mRNA and proteins***

Intra-striatal infusion of 6-OHDA induced a significant increase in Bcl-2 mRNA (*P*<0.001; Figure 5A) and its protein (*P*<0.01; Figure 5D) expression in the striatum compared to rats in the sham group. Moreover, exposure to SD decreased striatal Bcl-2 mRNA (*P*<0. 001; Figure 5A) and its protein (*P*<0. 001; Figure 5D) in the OH+SD group compared to the OH and OH+GC groups. 

As depicted in Figure 3B and E, intra-striatal injection of 6-OHDA did not induce a significant change in the Bax mRNA and protein levels. Furthermore, induction of SD elevated Bax expression both at mRNA (*P*<0. 01; Figure 5B) and protein (*P*<0. 001; Figure 5E) levels in OH+SD rats compared to the OH and OH+GC groups.

As shown in Figure 5C, induction of SD for 14 days significantly reduced Bcl-2/Bax mRNA (*P*<0. 001) and protein (Figure 5F; [*P*<0. 001]) ratios in comparison with the OH and OH+GC groups. No difference was found in the Bcl-2/Bax mRNA and protein in the striatum of OH+GC group compared to the OH group (Figure 5C and F).

***OFT***

The traveled distance was significantly lower in the Parkinsonian rats than that of the sham group 14 days post 6-OHDA-lesion induction (*P*<0.001). Also, induction of SD significantly increased total distance movement in the OH+SD group compared to the OH and OH+GC groups (*P*<0.05). The traveled distance did not significantly change between OH+GC and OH groups (Figure 6).

## Discussion

This study showed that exposure to long-term (14 days) SD could attenuate the anti-apoptotic resistance in the 6-OHDA-lesioned rats. We also found that SD relatively obscured motor impairment observed in OFT. Moreover, our results showed that the intra-striatal injection of 6-OHDA (10 μg/rat) resulted in the activation of anti-apoptotic compensatory responses to prevent apoptotic cell death in nigrostriatal pathway. However, these reactions were not completely able to mask motor abnormalities in the Parkinsonian rats. 

Interestingly, the disruptive effect of SD on the anti-apoptotic compensatory reaction of 6-OHDA-lesioned rats seems to be due to SD alone rather than the stress of the SD technique, because rats in the OH+GC group did not show any changes at the molecular and behavioral levels compared to the OH group. The unilateral 6-OHDA rat model of PD provides quantifiable motor disturbances, and when neurotoxin is injected into the striatum, it results in the retrograde degeneration of the nigrostriatal neurons (36, 37). Therefore, partial lesion of striatum by 6-OHDA induces a gradual degeneration of DAergic neurons and depletion of striatal DA levels. This makes it an ideal model to mimic early stages of the disease (36).

Our results showed that 6-OHDA lesion increased BDNF content in the striatum. This is in accordance with the previous studies that have demonstrated an increased level of BDNF in PD patients and in the midbrain of 6-OHDA-lesioned rats (7, 38, 39). Because BDNF is abundantly distributed in the DAergic neurons of the nigrostriatal pathway (40), the destruction of DAergic neurons should diminish BDNF expression. Therefore, an elevation in BDNF levels still remains a matter of controversy. However, following 6-OHDA infusion, both astrocytes and microglial cells proliferate and release BDNF, which may enhance signaling in the remaining DAergic neurons (7, 38, 41). 

Neurotrophic factors including BDNF are extensively distributed in basal ganglia and provide trophic supports to the motor neurons (42). BDNF binding to the tropomyosin receptor kinase B (TrkB) receptors in the striatum and substantia nigra triggers PI3K/Akt and MAPK/ERK signaling cascades (43). Activation of these pathways inhibits apoptosis, and induces neurite extension and neurogenesis in DAergic neurons (44). The present study suggests that partial 6-OHDA lesion to the striatum may provoke positive compensatory actions to maintain the functional integrity of DA neurons in nigrostriatal pathway, which is in accordance with previous report (45). Moreover, normal DAergic neurotransmission is in part modulated by BDNF insofar as it regulates DA turnover and induces striatal DA release in normal and pathologic conditions (46, 47). Our results also showed that SD reduced the increased levels of BDNF in 6-OHDA-lesioned rats. 

There is a close relationship between BDNF and sleep regulation, because exogenous BDNF increases sleep duration in different animal species, and SD reduces brain BDNF levels (48). 

According to Guzman-Marin *et al*., SD decreases BDNF expression in the hippocampus of the rats (49). Also, it has been shown that stimulation-provoked increase in the hippocampal BDNF content is suppressed by SD (50). BDNF and cAMP response-element-binding protein (CREB) have reciprocal effect on each other. So that, BDNF activates CREB-related protein expression, and BDNF gene is targeted by CREB activity (51). As a result, BDNF synthesis can be induced under CREB effect (50). Because SD reduces brain expression of CREB (52), it may be postulated that SD reduces striatal BDNF through a mechanism involving reduction of CREB levels in the rats with 6-OHDA lesion.

Our results demonstrated that both Bcl-2 and Bax are expressed (at mRNA and protein levels) in the striatum of sham rats. Apoptosis is mediated through two distinct members of Bcl-2 family including Bax and Bcl-2. The Bax protein facilitates the release of apoptogenic molecules from the mitochondria. Opposed to Bax, Bcl-2 proteins block mitochondrial leakage of cytochrome c through stabilizing mitochondrial outer membrane and prevent apoptotic cell death (9, 53, 54). In this study, we showed that Bcl-2 mRNA and its protein levels increased in the 6-OHDA-lesioned rats. *In vitro* findings showed that elevation of Bcl-2 expression following exposure to 6-OHDA reduces apoptosis and enhances cells viability (23). Moreover, increased Bcl-2 expression was reported in the post-mortem brains of the patients with PD, which has been noted as an anti-apoptotic reaction of remaining neurons. Similar results have been achieved with studies on DAergic neuronal injury induced by chronic neuroleptic regimen in the rat model (55). Because Bcl-2 promotes cell survival, an up-regulation of Bcl-2 may reflect a compensatory reaction of the un-affected neurons to prevent neuronal injuries (55).

We found that Bax mRNA and its protein levels remain unchanged after 14 days in the 6-OHDA-lesioned rats. This is in line with the reports that showed no alteration in the expression levels of Bax protein in the post-mortem brain tissue of the PD patients (56). Because Bax expression pattern is time-dependent and changes dynamically in the neuronal cells during the course of the chronic interventions (55), expression pattern of Bax upon different insults is very complex. 

In this study, a reduction in the elevated level of striatal Bcl-2 mRNA and protein induced by SD was associated with an increased level of Bax both at mRNA and protein levels in the Parkinsonian rats. Because the ratio of these two members predicts the cells’ tendency to apoptosis, it is probable that SD may cause an imbalance of the Bcl-2/Bax ratio that tilts the scales toward DAergic neurons degeneration in the OH+SD rats.

Immunohistochemical investigations on the rat brain revealed that SD reduces Bcl-2 positive neurons and increases the number of Bax expressing neurons, resulting in the neuronal loss via apoptotic pathways (57). SD has a negative impact on the cytoskeletal proteins including actin and tubulin. This in part alters the shape and size of neuronal cells (58). On the other hand, it has been revealed that impairment of the cytoskeletal proteins may trigger induction of neuronal apoptosis through disruption of mitochondrial position and reduction of anti-apoptotic activity of Bcl-2 (59). 

Given these, one possible mechanism for reduction of the striatal Bcl-2 levels may be the disruptive effect of SD on the cytoskeletal proteins finally leading to the apoptotic cell death.

Moreover, the expression of Bcl-2 protein is in part modulated by BDNF. Almeida *et al*. showed that incubation of the hippocampal neurons in the BDNF-enriched media for 24 hr increases Bcl-2 expression level (60). Presumably, this neuroprotective effect is mediated through PI3-K/Akt signaling pathway that induces expression of pro-surviving proteins by the activation of CREB and nuclear factor-kB (61). As noted previously, SD reduces BDNF levels and negatively affects its survival-promoting function. 

An increase in the striatal Bax protein following SD in the Parkinsonian rats can be attributed to the dysregulation of PI3K/AKT pathway. Akt phosphorylation by Ca^2+^ decreases the level of pro-apoptotic Bcl-2-associated death promoter (BAD) protein (58). BAD facilitates apoptosis by attaching to the anti-apoptotic proteins and thus blocking their survival-inducing properties. In normal condition, Bcl-2 and Bax proteins interact with each other to form a complex that inhibits apoptosis (62). However, by binding to this complex, BAD may displace Bax from Bcl-2 resulting in the cytosolic accumulation of Bax and promotion of cell death (63). Then, Bax rapidly translocates to the mitochondria and triggers apoptosis via release of cytochrome c (64). As a study by Somarajan *et al*. showed, SD prevents Akt phosphorylation through reduction of Ca^2+^ influx, which ultimately leads to the elevation of pro-apoptotic proteins and their subsequent apoptogenic activities (58). In our study, assessment of locomotor performance using OFT showed that Parkinsonian rats traveled shorter distance than sham rats. In agreement with the previous reports, this finding indicates that 6-OHDA is able to induce hypokinetic motor behavior in rats (65). This reduction in the motor activity reveals that the effectiveness of activated compensatory responses is not enough to cease motor deficits (at least at the level OFT sensitivity) in the 6-OHDA-lesioned rats. On the other hand, total-travelled distance in open field was higher in the Parkinsonian rats upon 14 days SD in comparison with the 6-OHDA-lesioned rats. In accordance with this, Dos Santos *et al*. reported that reduction in the locomotor activity of the Parkinsonian rats is averted by the induction of SD for 22 consecutive days. Also, their findings showed that SD not only elevates DA levels but also increases its turnover (66). Neuroimaging study using specific radio-ligands for DA receptors and transporters in human cases showed that SD increases striatal DA levels possibly through an increase in the DAergic neurons firing and DA release into the synaptic space (67). The activity of suprachiasmatic nucleus for increasing DA tone in the striatum may be responsible for its wakefulness-promoting effects (68). This nucleus modulates striatal DA levels through thalamo-striatal pathways and direct projections into mesencephalic DA neurons (69). Therefore, an increase in the nigrostriatal DA transmission might reflect a compensatory response to oppose DA degradation for retaining wakefulness and reduction of sleep drive after chronic period of SD (66, 67). It has been suggested that increased synaptic levels of DA and its turnover induce oxidative stress due to increased DA metabolism and autoxidation to reactive metabolites (70). Nevertheless, the striatum receives massive DAergic inputs in the brain, and DA neurons are more vulnerable to oxidative stress (71), which can predispose DAergic neurons to apoptosis (72). Therefore, an increased burden of oxidative stress due to SD may suppress anti-apoptotic responses in the 6-OHDA-lesioned rats. 

## Conclusion

SD suppressed the anti-apoptotic compensatory responses in the striatum. Therefore, it may accelerate continual neuronal cell death in PD. Given the fact that sleep disorders are frequently experienced by PD patients, management of this complication may slow down progressive DAergic neuronal loss. Due to complex neuronal effects of SD, further preclinical and clinical studies should be designed to find out its exact role in PD.
